# The Big Picture: Evidence Base and Current Trials in Cardiac CT

**DOI:** 10.1007/s40134-013-0022-z

**Published:** 2013-09-04

**Authors:** Holger Hetterich, Konstantin Nikolaou, Maximilian F. Reiser, Fabian Bamberg

**Affiliations:** 1 Department of Clinical Radiology, Ludwig Maximilians University of Munich, Marchioninistrasse 15, 81377 Munich, Germany; 2 DZHK (German Centre for Cardiovascular Research), partner site Munich Heart Alliance, Munich, Germany

**Keywords:** Cardiac CT, Acute coronary syndrome, Stable coronary artery disease, Prognosis, Clinical trials

## Abstract

Cardiac computed tomography angiography (CCTA) has technically matured into a robust imaging modality for various cardiac disorders. Whereas early trials focused on assessment of the efficacy of CCTA in comparison with established recommended methods, current research efforts focus on the effectiveness of the technique in specific clinical scenarios. In this article, we provide an overview of recent technology advances, describe major clinical scenarios in which CCTA has been evaluated, and detail pertinent evidence from completed or ongoing clinical trials, including its use to investigate acute chest pain, its use among patients with stable chest pain syndrome, and its prognostic value for the occurrence of cardiovascular events.

## Introduction

Over the last decade cardiac computed tomography angiography (CCTA) has emerged as an established technique for assessment of coronary artery disease (CAD) [1]. As a result of major technical developments, for example multi-slice and dual source scanners, prospective electrocardiogram (ECG) triggering, tube current modulation, lower tube voltage procedures, and advanced post-processing techniques, the technique has matured into a clinically useful modality with residual associated side effects, including low radiation exposure and contrast administration [2–5]. As a result, CCTA is now a fast, safe, and robust imaging modality for various cardiac disorders [1].

Whereas early trials focused on assessment of the efficacy of CCTA in comparison with established reference methods, for example invasive coronary angiography or intravascular ultrasound, more recently, the effectiveness of the technique in specific clinical scenarios has been under investigation. These studies address the true value of the technique in a real-world clinical setting and identify potential downstream effects, for example increased test utilization and costs.

In this review, we provide an overview of recent technological advances, with special emphasis on radiation exposure, describe major clinical scenarios in which CCTA has been evaluated, and detail pertinent evidence from completed or ongoing clinical trials, including its use to investigate acute chest pain, use of CCTA for patients with stable chest pain syndrome, and its prognostic value for the occurrence of cardiovascular events.

## Recent Technical Advances of CCTA

Current implementation and evaluation of CCTA in a variety of clinical scenarios can predominantly be attributed to substantial technical developments in recent years resulting in short examination times, high temporal and spatial resolution, and low-dose acquisition techniques.

Initially, CCTA was performed using retrospective ECG gating applying continuous photon emission throughout the cardiac cycle. However, although this approach allows for substantial flexibility with regard to post-processing and assessment of left ventricular function, radiation exposure usually totals 10–20 mSv [6]. More recently, prospective ECG triggering has been widely adopted; in this, photon emission is applied discontinuously over the cardiac cycle, resulting in an average radiation exposure of ~3.5 mSv [6], comparable to the annual background radiation in the general population [7]. Latest scanner generations enable use of high-pitch acquisition procedures (pitch values >3) as a result of angulated detector installations, which result in dramatically reduced radiation exposure of <1 mSv and enable scanning within a single heart beat in patients with slow sinus rhythm [8].

At the same time, advanced reconstruction techniques with or without the use of low tube current procedures have been shown to lead to lower radiation exposure acquisitions with maintained high image quality [9]. Overall, it is widely accepted that because of these advances, CCTA technology has a risk profile within the lower range of radiation exposure indexes of established CAD imaging modalities, for example nuclear perfusion imaging [10]. However, given the generally higher risk of radiation in distinct subpopulations (younger patients and females) [11], careful weighting of potential diagnostic benefits in each clinical setting is mandatory. As such, the evidence of the benefit of CCTA is currently under extensive investigation.

## Efficacy of CCTA for Significant Coronary Stenosis

After several small, single-center studies, three major, multicenter trials studied the diagnostic accuracy of CCTA for detection of significant coronary stenosis in comparison with invasive angiography, the established clinical reference standard [12–14]. They uniformly show that CCTA is able to achieve very high sensitivity and negative predictive value (both >95 %) and that its specificity and positive predictive value is moderate to high. Theoretically, on the basis of Bays’ theorem, these characteristics result in most useful application in low to intermediate prevalence populations, which are primarily encountered in the acute and stable chest pain setting [15, 16]. Among these populations, the high sensitivity and negative predictive value lead to substantial downward revision of probability; this enables safe rule-out of the disease of concern.

## CCTA in Acute Chest Pain

### The Clinical Challenge

Acute chest pain is one of the leading symptoms in the emergency department (ED) with more than eight million visits annually in the United States alone [17]. Patients with symptoms suggestive of cardiac ischemia but normal initial ECG and biomarkers remain a diagnostic challenge [18, 19]. Although only a minority of such patients will finally be diagnosed with cardiac ischemia, most are admitted to the hospital to undergo serial ECG and troponin evaluation and additional stress testing [20, 21]. This approach limits diagnostic errors to a minimum but is time-consuming and expensive with associated cost of 10–12 billion dollars annually in the United States [22]. Despite this comprehensive approach 2–5 % of acute coronary syndromes are missed [23].

### Diagnostic Efficacy of CCTA

In several observational cohort studies, CCTA has been studied in comparison with invasive coronary angiography for detection of ACS. One of the major studies, the *Rule Out Myocardial Infarction/Ischemia Using Computer Assisted Tomography (ROMICAT)* study, found excellent sensitivity and negative predictive value (both 100 % for the presence of any atherosclerotic plaque) to rule out ACS in 368 subjects who presented with acute chest pain to the ED and in whom the initial clinical evaluation was inconclusive (normal or non-diagnostic ECG changes and cardiac serum biomarkers) [24]. Hollander et al. [25] examined 568 patients with potential ACS and a low Thrombolysis on Myocardial Infarction (TIMI) risk score. Eighty-four percent of these patients were discharged home after CCTA. For patients with a negative CCTA, no cardiovascular deaths or nonfatal myocardial infarction occurred. These and others efficacy studies [26, 27] have led to expectations of efficient and safe assessment of patients with acute chest pain and low to intermediate likelihood of CAD by use of CCTA.

### Effectiveness of CCTA

**Fig. 1:**
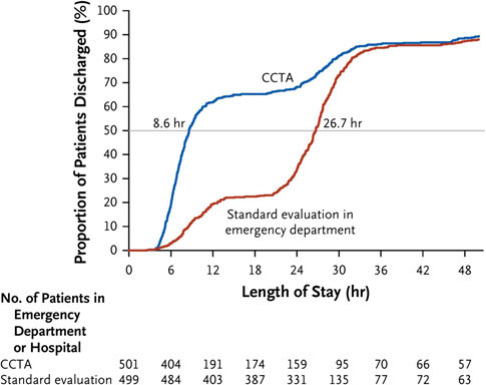
Length of stay in the hospital and proportion of patients discharged as observed in the ROMICAT II trial [29••]. There was a significant difference between observed discharged rate in the strategy implementing cardiac computed tomography angiography (CCTA) (*blue*) compared with standard evaluation in the emergency department (*red*), resulting in significantly lower median length of stay in the CT-based strategy. From Ref. [29••], with permission

As a result of encouraging efficacy studies, CCTA has been studied in three large multicenter randomized trials to determine whether implementation of the technique results in improved clinical outcome in the acute chest pain setting (Table 1). The *Coronary Computed Tomography Angiography for Systematic Triage of Acute Chest Pain Patients to Treatment (CT*-*STAT)* study was performed on 699 subjects and enrolled patients with acute chest pain but inconclusive evaluation in the ED [15]. Randomization was performed between a CT-based strategy versus a stress and rest myocardial perfusion imaging strategy. As a primary endpoint, a 54 % reduction in time-to-diagnosis was observed in the CCTA group. However, one criticism was that stress imaging alone might have sufficed in most cases to rule out obstructive CAD, which would have resulted in a shorter time to diagnosis [28]. As a follow-up trial of the observational cohort, the ROMICAT II trial was designed as a large, multicenter randomized trial that enrolled 1,000 patients with low to intermediate likelihood of ACS. The study was powered to a primary endpoint of length of stay in the hospital; secondary endpoints included time to diagnosis, rate of discharge from the ED, and resource utilization. Over a study period of 21 months, Hoffmann et al. [29••] found that patients in the CCTA group had shorter length-of-stay in the hospital (Fig. 1) and almost four times as many patients were discharged directly from the ED without hospital admission (47 vs. 12 %). The third large randomized multicenter randomized trial was performed among five centers in Pennsylvania [30]. Over a study period of 28 months, 908 patients with a low TIMI score of 0–2 were enrolled in a CCTA group and 462 received traditional care. The authors found that patients undergoing CT had a higher rate of discharge from the ED (50 vs. 23 %) and shorter length-of-stay in the hospital (difference 6.8 h) [30].

**Table 1:** Randomized multicenter trials evaluating the use of coronary computed tomography angiography for patients with acute chest pain

Study	Goldstein, CT-STAT, JACC		Hoffmann, ROMICAT II, NEJM		Litt, ACRIN AP, NEJM	
Year	2011		2012		2012	
CT scanner	64-slice or greater		64-slice or greater		64-slice or greater	
Number of patients in CT group	361		501		908	
Number of patients in control group	338		499		462	
Follow-up (months)	6		1		1	

Study	CT group	Control group	CT group	Control group	CT group	Control group
Rate of direct discharge from ED in CT group	262/361 (72.6 %)	271/338 (80.2 %)	233/501 (46.5 %)	62/499 (12.4 %)	450/908 (49.6 %)	105/462 (22.7 %)
Time-to-diagnosis in CT group (mean ± SD) (h)	2.9 ± 2	6.2 ± 7	10.4 ± 12.6	18.7 ± 11.8	n.a.	n.a.
Costs of care in ED [mean ± SD (thousand dollars)]	2.1 ± 0.7	3.5 ± 0.7	2.1 ± 0.1^a^	2.5 ± 0.1^a^	n.a.	n.a.
Rate of ED re-admission	2/361 (0.6 %)	4/338 (1.1 %)	14/501 (2.8 %)	19/499 (3.8 %)	71/885 (8.0 %)	34/452 (7.5 %)

*CT* computed tomography, *ED* emergency department, *SD* standard deviation, *n.a.* not available

^a^Costs of care relate to a subset of 649 patients

Notably, this effect is associated with an excellent safety profile of CCTA. In two large studies there was no myocardial infarction or cardiac death within the first month after a negative CCTA scan [29••, 30]. Furthermore, the ROMICAT and CT-STAT showed that the absence of significant coronary atherosclerosis is associated with the absence or a minimum number of major acute coronary event (MACE) in 6 months of follow-up [24, 31].

### Ongoing Large Clinical Trials

The *Better Evaluation of Acute Chest Pain With Computed Tomography Angiography* (BEACON) study is a randomized, controlled trial, currently being conducted in the Netherlands, evaluating the incremental value of CCTA among approximately 500 patients with acute chest pain and suspected ACS (*ClinicalTrials.gov Identifier: NCT01413282*). The study is evaluating successful discharge rate of patients undergoing CCTA. Furthermore, in contrast with other studies, the investigators also intend to examine the positive predictive value of severe CAD defined by CCTA for subsequent necessary revascularization. Because this study is the first European trial examining the use of CCTA in the ED setting, the results are relevant, because they provide initial findings on potential benefits in healthcare costs and resource allocation outside the US healthcare system.

The Study *Comparing CT Scan and Stress Test in Patients With Known Coronary Artery Disease Hospitalized for Chest Pain (PROSPECT*-*CAD)* focuses on patients hospitalized for acute chest pain, comparing CCTA and radionuclide stress myocardial perfusion imaging (*ClinicalTrials.gov Identifier: NCT01106612*). The study objective is to determine whether CCTA or nuclear stress testing is superior in identifying chest pain patients with severe CAD who need to undergo revascularization according to findings of invasive angiography and current guidelines.

### Costs Associated with CCTA

The CT-STAT trial suggested a potential reduction in costs of care by 38 % when CCTA was compared with myocardial perfusion imaging [31]. However, these results were not derived from formal cost-effectiveness analysis nor were downstream costs included in the analysis [31]. In a more comprehensive approach the ROMICAT II trial analyzed average costs of care among a subgroup of 649 patients from five centers from the initial visit through the 28-day follow-up period. Although there was a reduction of cost in the ED, hospital costs were increased in the CCTA group compared with the standard of care because of additional testing [29••]. Combined costs from ED and hospital were similar in both groups. There are a limited number of formal cost-effectiveness analyses that indicate that the incremental cost-effectiveness ratio is highest in subpopulations, e.g. younger women [15].

Overall, it seems that further, more cost-oriented analysis is warranted within large randomized trials, to determine whether use of CCTA among patients with acute chest pain eventually results in increased test utilization with limited benefit for the patient. Also, available cost estimates are only true for the United States and have not been evaluated for other countries where reimbursement might be substantially different for imaging, laboratory and other diagnostic testing.

## CCTA in Stable Angina Syndromes

### The Clinical Challenge

The diagnostic work-up of patients with suspected significant CAD depends on the pretest likelihood of disease, which can be assessed by use of a variety of scoring systems, e.g. the Diamond–Forrester Classification [32, 33]. Established tests include stress ECG, echocardiography, and myocardial perfusion imaging [34]. However, despite these tools, most invasive angiograms remain purely diagnostic and are associated with significant side effects, including complications at the femoral puncture site [35, 36]. As such, a non-invasive, accurate tool to rule out the presence of significant coronary artery stenosis which may eliminate the need for invasive angiography may substantially affect patient management. Given its diagnostic accuracy with high sensitivity and negative predictive value, CCTA has been increasingly recognized as a valuable tool among patients with low to intermediate likelihood and stable angina [1].

### Efficacy of CCTA

Most of the initial studies on the diagnostic accuracy of CCTA were obtained from patients referred for invasive angiography who presented with stable angina. Two recent meta-analyses pooled data from 960 and 1,286 patients, respectively [37, 38]. Both analyses found an excellent sensitivity of 99 % for detection of coronary artery stenosis defined as >50 % luminal narrowing in comparison with quantitative coronary angiography. The *Assessment by Coronary Computed Tomography Angiography of Individuals Undergoing Invasive Coronary Angiography (ACCURACY)* study, which included 230 patients, was the first prospective multicenter trial to evaluate stable patients without prior known CAD undergoing CCTA and quantitative coronary angiography [12]. The study found sensitivity of 95 %, specificity of 83 %, a positive predictive value of 64 %, and a negative predictive value of 99 %. Notably, these results are based on evaluation of all vessel segments and patients were not excluded because of heart rate, body mass index, or coronary artery calcium (CAC) score [12].

### Effectiveness of CCTA

Data from reasonably sized randomized controlled trials of CCTA in the setting of stable angina are not yet available. As such, recommendations by current appropriate use criteria for CCTA among patients with stable angina but low-to-intermediate CAD likelihood, especially in the setting of equivocal prior study results, are based on observational studies [1]. One of the largest observational trials in this context is the *Coronary Artery Evaluation Using 64*-*Row Multidetector Computed Tomography Angiography (CorE*-*64)*, an international, multicenter study. Both pretest probability and CAC scoring were important factors for the effectiveness of CCTA to detect significant coronary stenosis, because CCTA was found to be less effective among patients with a CAC score of ≥600 and those with a high pretest probability for obstructive vessel disease [13].

### Ongoing Large Clinical Trials

While existing evidence is scarce, there are two large ongoing randomized studies of patients with stable chest pain syndrome.

The *PROspective Multicenter Imaging Study for Evaluation of Chest Pain (PROMISE)* is a randomized controlled trial to determine whether an initial non-invasive anatomic imaging strategy with CCTA is associated with improved clinical outcomes in comparison with a functional testing strategy, including exercise ECG, stress echocardiography, and nuclear myocardial perfusion imaging (*ClinicalTrials.gov Identifier: NCT01174550*). Primary and secondary endpoints include time to first event, death, MACE, cumulative radiation exposure, medical costs, and quality of life. The study intends to include 10,000 patients with more than 9,000 subjects enrolled in July 2013.

The *Randomized Evaluation of Patients With Stable Angina Comparing Diagnostic Examinations (RESCUE)* is a randomized, controlled multicenter trial to compare a CCTA-based strategy with a nuclear myocardial perfusion-based diagnostic strategy among patients with stable angina (*ClinicalTrials.gov Identifier: NCT01262625*). Results from CCTA and myocardial perfusion imaging are used to direct patients to follow-up visits, optimum medical therapy, and revascularization. Over the follow-up period, incidence of MACE as a primary endpoint is documented. The study is intended to enroll 4,500 patients. The main study hypotheses are that use of CCTA is associated with no increase in MACE or revascularization, reduced cost, reduced risks, additional insights into alternate explanations of chest pain, and increased cost-effectiveness.

The excellent diagnostic performance and safety profile of CCTA enables its use as a reference in large-scale clinical trials. The purpose of the *International Study of Comparative Health Effectiveness With Medical and Invasive Approaches (ISCHEMIA)* is to evaluate the best treatment strategy for patients with stable ischemic heart disease and high risk profile (*ClinicalTrials.gov Identifier: NCT01471522).* The study is intended to enroll 8,000 patients who will randomly be assigned to one of two treatment groups: routine invasive coronary angiography followed by revascularization plus optimum medical therapy or optimum medical therapy alone, with revascularization reserved for those who fail medical treatment. The study is powered for a primary endpoint of cardiovascular death or nonfatal myocardial infarction. Before randomization, patients will undergo CCTA to exclude subjects without obstructive CAD or with unprotected left main disease.

### Costs Associated with CCTA

Several cost-analyses are available demonstrating that, for patients with a low to intermediate likelihood of obstructive CAD, CCTA is particularly cost-effective compared with other imaging modalities, for example myocardial perfusion imaging [39, 40]. When modeling a CT-based strategy, adjusted total healthcare costs were reduced by 27 % and disease-specific costs were reduced by 33 % compared with myocardial perfusion imaging [39]. In a comprehensive cost-effectiveness model by Ladapo et al. CCTA was associated with an increase of overall costs because of increased detection of CAD and partly because of follow-up of incidental findings. However, parts of these costs are offset by lower costs of care for myocardial infarction and stroke. In comparison with the least effective test, use of CCTA reduced adverse event rates by 3 % in men and women [41]. When performed with stress testing, the incremental cost-effectiveness ratio of CCTA ranges from $26,200 per quality-adjusted life-year for men to $35,000 per quality-adjusted life-year for women, and is within the range generally regarded as cost-effective [41].

## Prognostic Value of CCTA

### The Clinical Challenge

Besides the diagnosis of overt cardiovascular disease state, risk stratification remains of critical importance to guide lifestyle modification and therapeutic options. Established imaging modalities that encompass prognostic information are myocardial perfusion imaging and stress echocardiography [42], which have also shown to be useful in patient management. CCTA has the potential to provide information on coronary morphology and disease burden that is currently not available from any other non-invasive imaging technology, including presence and extent of calcified, non-calcified, and mixed atherosclerotic plaque at no extra costs (radiation or contrast administration) [43, 44]. However, until recently there were few results indicating whether or not this information enables determination of prognosis.

### Prognostic Evidence

Pooling the evidence from several smaller single-center studies, a recent meta-analysis summarized the prognostic significance of CCTA findings for MACE among patients with stable chest pain syndromes [45]. Data from a total of 7,335 patients in 11 single studies meeting rigorous inclusion criteria were included. The analyses indicated that most of the findings of CCTA, including the presence and number of significant coronary stenoses and the presence, type, and extent of non-significant plaque are strong independent predictors of cardiovascular events. For example, the presence of one or more significant coronary stenosis was associated with an annualized event rate of 11.9 % and a hazard ratio of 10.74 [45]. Lately, the *Coronary CT Angiography Evaluation for Clinical Outcomes: An International Multicenter Registry (CONFIRM)* as a large multinational prospective, observational registry of patients undergoing CCTA included more than 27,000 patients from twelve cluster sites in six countries in Europe, North America, and Asia [46, 47]. The study includes symptomatic patients with suspected CAD, patients with known CAD, and asymptomatic individuals, who were followed after CCTA for the occurrence of MACE including death, myocardial infarction, unstable angina, revascularization, and hospitalization. Within this large sample, various aspects of the value of CCTA with a special emphasis on prognostic relevance were studied [46, 47].

**Fig. 2:**
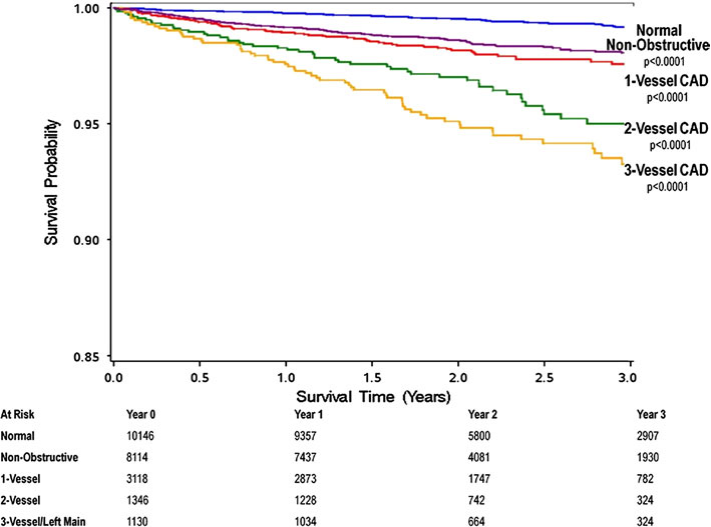
All-cause three-year survival according to the presence, extent, and severity of cardiac computed tomography findings from the CONFIRM sample [49••]. A dose–response relationship of mortality with increasing numbers of vessels with obstructive coronary artery disease (CAD) was observed. From Ref. [49••], with permission

A major finding of CONFIRM was that the absence of any coronary atherosclerosis is associated with an excellent prognosis, with all-cause mortality as low as 0.65 % after a mean follow-up of 22.5 months. Among patients with CAD, mortality increases from 1.99 % for patients with non-obstructive CAD to 4.95 % for patients with high-risk obstruction [48]. Furthermore there is a dose–response relationship between the number of vessels with obstructive CAD and all-cause mortality, with increasing risk among patients with single-vessel disease (HR 2.00), two-vessel disease (HR 2.92), and three-vessel or left main disease (HR 3.70) [49••] (Fig. 2). Given the large size of the CONFIRM sample the investigators were also able to examine outcomes of treatment strategies for patients with different extent of CAD, as defined by CCTA. Revascularization was associated with a survival benefit among patients with high-risk CAD but not in patients without the high-risk pattern [50]. Interestingly, the CONFIRM investigators found relatively high rates of non-obstructive and obstructive CAD in patients with a CAC score of zero (13.5 and 3.5 %, respectively) which was associated with increased cardiovascular events [51].

## Conclusions

CCTA has matured into a robust technique with the potential to enhance the work-up of patients in a variety of clinical settings. The volume of evidence is continuously increasing and evaluation of the efficacy of CCTA compared with established recommended methods for detection of significant coronary stenosis has moved beyond scientific evaluation. Instead, current efforts focus on the real-world value and effectiveness of the technique in two major scenarios:

1. patients with acute chest pain; and
2. patients presenting with symptoms suggestive of stable angina.

As the next step in assessment of this novel technology, these large-scale studies are designed as randomized clinical trials in which one group incorporates CCTA as the diagnostic intervention. These trials are critical, because they identify associated side effects, acceptance, and costs. Such studies, alone, will provide the scientific basis for recommendations by international societies and health care providers and affect reimbursement procedures.

In the setting of acute chest pain, most of the evidence has been obtained from three large randomized diagnostic trials; these also indicate that use and costs of CCTA require further research. In contrast, such evidence is scarce in the setting of stable angina and current recommendations are based on observational studies and derived cost-effectiveness analysis. However, there are three major ongoing trials that will furnish more comprehensive data and are expected to provide sufficient evidence to enable evaluation of the role of CCTA among patients with stable chest pain (PROMISE, RESCUE, and ISCHEMIA). There is rapidly emerging evidence of the prognostic value of the findings of CCTA for the occurrence of cardiovascular events and it can be predicted that the prognostic relevance of CCTA findings will complement the diagnostic value of the technique pending future studies; however, randomized trials are pending. Although stepwise evaluation of novel emerging imaging techniques is a resource-demanding, labor-intensive process, it is the only way costly imaging procedures can prove their superiority not only in respect of excellent image quality but also in respect of relevant outcomes in real-world scenarios. This approach, alone, will ensure that CCTA will be widely adopted clinically and will result in better management of our patients.
